# The prevalence of antimicrobial resistance in clinical isolates from Gulf Corporation Council countries

**DOI:** 10.1186/2047-2994-1-26

**Published:** 2012-07-19

**Authors:** Mahmoud Aly, Hanan H Balkhy

**Affiliations:** 1 King Abdullah International Medical Research Center, Riyadh, Saudi Arabia; 2 Infection Prevention and Control Department, King Abdulaziz Medical City, Riyadh, Saudi Arabia; 3 King Saud bin Abdulaziz University for Health Sciences, Riyadh, Saudi Arabia

**Keywords:** Antibiotics/antimicrobials, Resistance, GCC, (Saudi Arabia, Qatar, Bahrain, Kuwait, Oman, and United Arab Emirates) Gram negative, Gram positive, Anaerobes, Pathogens, Infection, Resistance mechanisms, Molecular typing

## Abstract

**Background:**

The burden of antimicrobial resistance worldwide is substantial and is likely to grow. Many factors play a role in the emergence of resistance. These resistance mechanisms may be encoded on transferable genes, which facilitate the spread of resistance between bacterial strains of the same and/or different species. Other resistance mechanisms may be due to alterations in the chromosomal DNA which enables the bacteria to withstand the environment and multiply. Many, if not most, of the Gulf Corporation Council (GCC) countries do not have clear guidelines for antimicrobial use, and lack policies for restricting and auditing antimicrobial prescriptions.

**Objective:**

The aim of this study is to review the prevalence of antibiotic resistance in GCC countries and explore the reasons for antibiotic resistance in the region.

**Methodology:**

The PubMed database was searched using the following key words: antimicrobial resistance, antibiotic stewardship, prevalence, epidemiology, mechanism of resistance, and GCC country (Saudi Arabia, Qatar, Bahrain, Kuwait, Oman, and United Arab Emirates).

**Results:**

From January1990 through April 2011, there were 45 articles published reviewing antibiotic resistance in the GCC countries. Among all the GCC countries, 37,295 bacterial isolates were studied for antimicrobial resistance. The most prevalent microorganism was *Escherichia coli* (10,073/44%), followed by *Klebsiella pneumoniae* (4,709/20%), *Pseudomonas aeruginosa* (4,287/18.7%), MRSA (1,216/5.4%), *Acinetobacter* (1,061/5%), with *C. difficile* and *Enterococcus* representing less than 1%.

**Conclusion:**

In the last 2 decades, *E. coli* followed *by Klebsiella pneumoniae* were the most prevalent reported microorganisms by GCC countries with resistance data*.*

## Findings

The burden of antimicrobial resistance worldwide is substantial and is likely to grow [1]. Furthermore, many factors play a role in the emergence of resistance such as from poor utilization of antimicrobial agents, transmission of resistant bacteria from patient to patient and from Health-care workers (HCWs) to patients and vice versa, lack of guidelines for appropriate and judicious use of antimicrobial agents and lack of easy-to-use auditing tools for restriction. In addition, there is clear misuse of antimicrobial agents in the animal industry, and most agents are the same agents used in humans. Further, there are few antimicrobial agents in the pipeline of production, leaving clinicians with minimal tools to combat these infections. All these factors, together, have led to the inevitable emergence and rise of resistance.

Bacteria develop antimicrobial resistance through many mechanisms including mutations in penicillin binding proteins, efflux mechanisms, alterations in outer membrane proteins and the production of hydrolyzing enzymes such as extended spectrum β lactamase (ESBL) and carbapenemases [2]. These resistance mechanisms may be encoded on transferable genes which facilitate the spread between bacteria of the same species and between different species. Other resistance mechanisms may be due to alterations in the chromosomal DNA which enables the bacteria to withstand the harsh environment and multiply.

Many, if not most, of the Gulf Corporation Council (GCC) countries do not have clear guidelines for antimicrobial use and lack policies for restricting and auditing antimicrobial prescriptions. There are no guidelines for the use of antimicrobials in the animal industries either. Thus, it is not surprising that antimicrobial resistance has emerged in these countries [3]. There are few reports studying prevalence rates of resistance among the different pathogens and mechanisms of resistance at the national level.

### Objective

The aim of this study is to review the prevalence of antibiotic resistance in GCC countries and explore the reasons for antibiotic resistance in the region.

### Methodology

The PubMed database was searched using the following key words: antimicrobial resistance, antibiotic stewardship, prevalence, epidemiology, mechanism of resistance, and GCC country (Saudi Arabia, Qatar, Bahrain, Kuwait, Oman, and United Arab Emirates). Specific organisms were searched for Gram negative bacteria: *Acinetobacter, Klebsiella pneumoniae, Pseudomonas aeruginosa* and *Escherichia coli,* and for Gram positive bacteria: *Methicillin-resistant Staphylococcus aureus (MRSA), Vancomycin-Resistant Enterococci (VRE)* and *Clostridium difficile (C. difficile).* To identify the resistance mechanisms, these studies followed routine laboratory diagnostics and surveillance testing.

### Statistical analysis

Statistical data were generated and analyzed using the statistical software SPSS version 19. Descriptive statistics were calculated and the weighted mean was estimated using the estimated marginal mean function in SPSS. Data were expressed as number (*n*) and percentage (%); Table 1. Records of the total numbers of clinical isolates were grouped based on their country and species according to their gram staining classification; Table 2. Data were represented as total number of isolates (*n*) and their percentage (%).

**Table 1:** The data of selected clinical isolates reported by GCC countries

**Country**	**Population**	**Population %**	**Reports (*n*)**	**Reports%**	**Isolates (*n*)**	**Isolates%**	**References**
Bahrain	1,106,509	2.9%	3	9.1%	2841	7.6%	[4-6]
Kuwait	2,583,020	6.7%	9	27.3%	20339	54.5%	[7-15]
Oman	3,173,917	8.2%	3	9.1%	882	2.4%	[8,16,17]
Qatar	1,608,903	4.2%	2	6.1%	570	1.5%	[7,18]
Saudi Arabia	25,373,512	65.7%	14	42.4%	12174	32.6%	[19-32]
UAE	4,765,000	12.3%	2	6.1%	491	1.3%	[33,34]
GCC total	38,610,861	100.0%	33	100.0%	37295	100.0%	*n =* 33 articles

GCC = Gulf Corporation Council; UAE = United Arab Emirates.

**Table 2:** The prevalence of resistant pathogens in clinical isolates from GCC countries

**Country**	**Gram Negative**				**Gram Positive**			
	***Acinetobacter***	***Escherichiacoli***	***Klebsiella pneumoniae***	***Pseudomonas aeruginosa***	***Clostridium difficile***	**Enterococcus**	**MRSA**	***Streptococcus pneumoniae***
**Bahrain**	N.R	14.0%	13.9%	N.R	N.R	76.5%	8.5%	N.R
**Kuwait**	16.7%	77.0%	36.2%	2.6%	70.0%	N.R	3.3%	66.3%
**Oman**	N.R	N.R	0.1%	0.3%	0.0%	N.R	58.3%	N.R
**Qatar**	N.R	1.1%	0.8%	0.6%	N.R	N.R	N.R	N.R
**Saudi Arabia**	83.3%	7.6%	48.3%	92.3%	30.0%	23.5%	29.9%	30.7%
**UAE**	N.R	0.3%	0.7%	4.2%	N.R	N.R	N.R	3.0%

GCC = Gulf Corporation Council; N.R = not reported; MRSA = *methicillin resistant Staphylococcus aureus*; UAE = United Arab Emirates.

## Results

**Figure 1:**
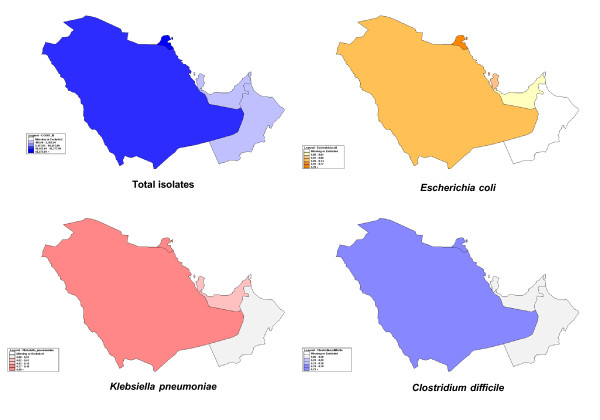
Geographical distributions of resistant isolates among GCC countries 1990–2011.

Between 1990 and April 2011, there were 45 articles published reviewing antibiotic resistance in GCC countries. Eleven articles were excluded because of their low number of isolates (*n* < 100) [35-45]. Thirty four articles were analyzed as they contained national data. Among all the GCC countries, 37,295 bacterial isolates were studied for antimicrobial resistance. The isolates were distributed as follows: Bahrain (2,841/7.6%), Kuwait (20,339/54.5%), Oman (882/2.4%), Qatar (570/1.5%), Saudi Arabia (12,174/32.6%) and UAE (491/1.3%); Figure 1.

The most prevalent microorganism was *Escherichia coli* (10073, 44%), followed by *Klebsiella pneumoniae* (4,709: 20%), *Pseudomonas aeruginosa* (4,287/18.7%), MRSA (1,216: 5.4%), *Acinetobacter* (1,061/5%), with *C. difficile* and *Enterococcus* representing less than 1%.

Some national isolates showed high resistance than others. In Qatar, Khan, Elshafie et al. 2010, reported the occurrence of resistant Gram-negative organisms in 63.1% of bacteremia patients (*n =* 452) with the following prevalences: ESBL-resistant *E coli* (34%), followed by *Klebsiella spp.* (39/13.7%) and finally *Pseudomonas aeruginosa* (7.4%) [18]. Shigidi and his group found that Staphylococcus was most prevalent (29%), followed by *E coli* (9%) and *Pseudomonas aeruginosa* was the least resistant with only 3% prevalence among dialysis patients in 2010 [7]. Their results, however, should be considered with caution because they tested relatively low number of cases (*n* = 118). A trend of *MRSA* infection in the burn center of the Sultanate of Oman was reported [16]. ESBL phenotypes were detected in more than 21% of the total isolates indicating their correlation with the resistance.

## Conclusions

The geographical distributions of the resistance isolates during (1990–2011) in the gulf region were shown in Figure 1*.* The most prevalent reported microorganism with resistance data reported by GCC countries in the last 2 decades was *E. coli,* followed by *Klebsiella pneumoniae* and others. Multiple ESBL genes were identified, however, no carbapenamases were present and thus further work to identify the mechanism of resistance, such as c-di-GMP and others, is required. Many hospitals within the Kingdom have identified carbapenem resistance among enterobacteriaceae, and Oman has reported NDM-1 in *Klebsiella* isolates in 2010[17]. From our experience at KAMC in Riyadh, a 1000 bed hospital, we have identified for the first time carbapenem-resistant *Klebsiella pneumoniae* which led to an outbreak stemming from the adult ICU [46].

The emergence of antimicrobial resistance in GCC countries might have occurred for several reasons. One is the readily available broad spectrum antibiotics, such as 3^rd^ and 4^th^ generation cephalosporins, quinolones and carbapenems in the healthcare settings. Most GCC countries lack the presence of antimicrobial stewardship programs, especially in the inpatient setting where broad spectrum antimicrobial agents are used. Most hospitals’ architectural designs are old and many harbor 2 and 4 bedded rooms, which do not allow for proper isolation of infected and colonized patients with multi-drug resistant organisms. There a lack of strong infection control programs, properly trained infectious disease specialists and clinical pharmacists in the field of infectious diseases. In advanced healthcare systems, integrated computerized programs allowing for the restriction of antimicrobial agents and providing decision-assisted physician orders have proven to be helpful in controlling the use of antibiotics. Such alternatives may be expensive and not suitable for all healthcare settings. Further, highly skilled and dedicated information technology personnel are needed to support these systems. Finally, the lack of clinical pharmacists and infectious disease specialists may be a major contributor to the current emergence of resistance. Multidisciplinary teams including: infectious diseases, intensivists, surgeons, pulmonologists and emergency room specialists and clinical pharmacists may be needed to enhance utilization of antibiotics and play an important role in recommending the appropriate antimicrobial regimens and proper guidance on treatment and de-escalation.

## Competing interests

The authors declare that they have no competing interests.

## Authors’ contributions

**MA:** acquisition, analysis and interpretation of data; drafting the manuscript. **HB:** analysis and interpretation of data; drafting the manuscript. All authors read and approved the final manuscript.
